# Low-Cost Nitric Oxide Sensors: Assessment of Temperature and Humidity Effects

**DOI:** 10.3390/s22229013

**Published:** 2022-11-21

**Authors:** Steven Owen, Lachlan H. Yee, Damien T. Maher

**Affiliations:** 1 Illawarra Coatings, 19 Technology Drive, Appin, NSW 2560, Australia; 2 Faculty of Science and Engineering, Southern Cross University, Military Road, Lismore, NSW 2480, Australia

**Keywords:** nitric oxide, air pollution, IoT, low-cost sensor, gas sensor, gas detection

## Abstract

High equipment cost is a significant entry barrier to research for small organizations in developing solutions to air pollution problems. Low-cost electrochemical sensors show sensitivity at parts-per-billion by volume mixing ratios but are subject to variation due to changing environmental conditions, in particular temperature. In this study, we demonstrate a low-cost Internet of Things (IoT)-based sensor system for nitric oxide analysis. The sensor system used a four-electrode electrochemical sensor exposed to a series of isothermal/isohume conditions. When deployed under these conditions, stable baseline responses were achieved, in contrast to ambient air conditions where temperature and humidity conditions may be variable. The interrelationship between working and auxiliary electrodes was linear within an environmental envelope of 20–40 °C and 30–80% relative humidity, with correlation coefficients from 0.9980 to 0.9999 when measured under isothermal/isohume conditions. These data enabled the determination of surface functions that describe the working to auxiliary electrode offsets and calibration curve gradients and intercepts. The linear and reproducible nature of individual calibration curves for stepwise nitric oxide (NO) additions under isothermal/isohume environments suggests the suitability of these sensors for applications aside from their role in air quality monitoring. Such applications would include nitric oxide kinetic studies for atmospheric applications or measurement of the potential biocatalytic activity of nitric oxide consuming enzymes in biocatalytic coatings, both of which currently employ high-capital-cost chemiluminescence detectors.

## 1. Introduction

Whilst it is now accepted that increased atmospheric pollutant concentrations are driving climate change, they also have a range of pronounced impacts on the environment and human health. Approximately 2.4 billion people reside in dwellings that use biomass fuel as the primary source of fuel for heating, cooking, or both. Estimates suggest that 3.2 million people die prematurely each year from illness directly attributable to the indoor air pollution generated from these biomass fuels [1]. A Taiwanese study in 2020 showed a high correlation between outpatient visits and increased concentrations of NO_2_, O_3_, particulate matter with a diameter ≤ 10 µm (PM_10_), CO_2_, and NO [2].

Oxides of nitrogen originating from combustion processes in internal combustion engines are found in the photochemically generated smog that envelopes many modern cities, along with O_3_. Therefore, monitoring these compounds is important to inform appropriate health advice and assess the effectiveness of management strategies to reduce air pollution.

Many techniques are available for measuring NO concentrations, ranging from simple diffusive samplers [3,4,5,6] to absorption and emission spectroscopy [7,8,9], gas chromatography [10,11,12], and mass spectroscopy [13,14]. Chemiluminescence, an example of an emission spectroscopy technique, is considered the standard for the measurement of nitrogen oxides for air quality monitoring [6,15,16,17,18,19]. Whilst capable of detecting nitrogen oxides at the parts per billion level (ppbV), drawbacks to the use of chemiluminescence detectors include the initial capital equipment cost (i.e., in the order of tens of thousands of US dollars per unit), ongoing maintenance and calibration costs, as well as the requirement for an ozone source for the oxidation reaction of NO to NO_2_.

The high capital cost of equipment is a significant entry barrier to research for small organizations in developing solutions to air pollution problems. This entry barrier and the steadily increasing concerns regarding the impact of air quality on human health have led to several commercial enterprises developing low-cost monitoring systems. Whilst these low-cost systems provide viable alternatives to high-cost government facilities for individuals to track their exposure, the accuracy and detection limits of these systems may not be suitable for all monitoring purposes [5].

Low-cost electrochemical sensors (LCESs) are electrochemical cells that, when exposed to the target gas, generate a current that is linearly proportional to the concentration of that gas. LCESs have been used for industrial safety applications since at least the 1970s [20]. LCES’s used for industrial safety applications typically measure target gases at the parts per million (ppm) level, whilst air quality monitoring is typically measured in the parts per billion (ppb) range, some 2–3 orders of magnitude lower than the industrial applications. Following improvements in the signal–noise ratio, sensitivity, and conditioning circuitry, Mead et al. demonstrated that LCESs can provide accurate measurements in the parts per billion (ppb) range under certain conditions [21]. Meyerhoff et al. compared a commercially available LCES with a chemiluminescence detector to measure the nitrite concentration in food samples and found a linear regression curve close to unity (R = 0.999) [22].

The drawback with these improved sensors thus far has been the response, particularly of NO sensors, to variations in ambient environmental conditions [21,23,24,25]. Significant effort has been made to develop methods to overcome these deficiencies, ranging from simple post-data corrections to machine learning algorithms [26,27,28].

Wei et al. [29] investigated the accuracy of LCESs under stable laboratory conditions and demonstrated the high potential of such sensors compared to conventional reference instruments. Wei et al. applied a linear correction methodology considering temperature and relative humidity, concluding that “careful data analysis and correction protocols are essential to guarantee good data quality” [29] (p. 73).

Wei et al. studied NO_2_, CO, NO and O_x_ sensors, whereas Rogulski et al. [30] focused solely on Alphasense NO_2_ sensors. finding that temperature affected the sensor accuracy more than relative humidity, but also that air temperatures > 30 °C could lead to absolute percentage errors > 150%. Thus, applying the correction methods recommended by Alphasense may not be appropriate under all environmental; conditions. The improved correction method proposed by Rogulski et al. [30] was based on a second-degree polynomial regression.

The main goal of our study was to assess the influences of temperature and humidity on an isolated low-cost NO electrochemical sensor and establish a polynomial surface function that combines the dependent variables of temperature and humidity for calibration of NO concentration. In contrast to previous studies, our focus was not on applying the derived functions to measure ambient air quality under changing environmental conditions. Rather, we focused on employing the sensors under isothermal/isohume conditions for applications in areas such as NO kinetic studies for atmospheric applications or measurement of potential biocatalytic activity of NO-consuming enzymes in biocatalytic coatings.

## 2. Materials and Methods

**Figure 1:**
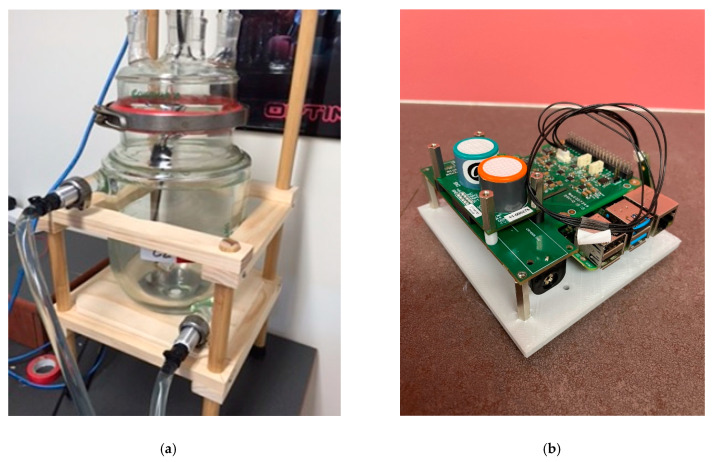
(**a**) Environmental chamber constructed from a Chemglass CG-1929-29 5-L double-walled reaction vessel fitted with a CG-1941 4-neck reaction vessel lid. (**b**) Low-cost electrochemical sensor showing Alphasense sensors attached to South Coast Science DFE.

A Chemglass Life Sciences CG-1929-29 5-L double-walled reaction vessel fitted with a CG-1941 4-neck reaction vessel lid was used as the atmospheric chamber. The reaction vessel was equipped with a ¾-inch beaded inlet/outlet pipe for water circulation between the vessel walls. The four inlet necks allowed a cable to power the Raspberry Pi and for inlet of zero air and nitric oxide gas. When assembled, the vessel had a total volume of 7.87 L (Figure 1a).

Chamber temperature was controlled by a Thermoline TU3 water circulator/heater-equipped water reservoir. Instrument-grade air from BOC Gases (Table 1) was used as a source of zero air for purging the reaction chamber.

Following the purge, humidity was first generated and then maintained using water/glycerol solutions according to ASTM D5036-2011. The use of water/glycerol solutions for generating and maintaining relative humidity conditions was preferred over gas bubblers due to the limitations in generating humidity’s greater than approximately 50–55% with gas bubbler systems [29]. Using this approach has demonstrated temperature and relative humidity stability of ±0.1 °C and ±0.5% relative humidity, respectively.

Once the desired temperature and humidity conditions were established, 60 ppm NO gas in nitrogen was injected into the chamber using a Swagelok VAF-G2-01M-1-0 Variable Area Flowmeter at a flow rate of approximately 1.0 L/h. NO gas flow was continued until the desired concentration was achieved.

The sensor system was constructed using an Alphasense Limited (Alphasense) NO NO-A4 mounted on an Alphasense analog front end (AFE). The AFE measured the electrode voltages from the working and auxiliary electrodes of the electrochemical sensor and provided an analog output of the values. The AFE was then connected to a South Coast Science (SCS) Alpha Pi Eng. Board (APEB) which conditioned the analog output of the Alphasense AFE and provided digitization of the electrode voltages through the use of ADS1115 analog to digital converters (ADCs). The voltage range of these converters is in the order of 2 volts (V) with 16-bit resolution, thereby providing a resolution of 3 × 10^−5^ V.

The APEB was equipped with a 40-pin header compatible with microcomputers such as the Raspberry Pi 3 and 4. A Raspberry Pi 4, 4GB was attached to the above system. Raspbian “Buster”, a Linux-based distribution, was used as the operating system, allowing a Microsoft Windows appearance to the microcomputer.

A Bosch BME280 sensor was attached to the 40-pin header to measure temperature and relative humidity. The BME280 is an integrated environmental sensor that has been specifically developed for applications such as home automation control, personalized weather stations, and sports fitness tools. The temperature sensor incorporated into the BME280 was calibrated against a National Institute of Standards and Technology (NIST)-calibrated thermometer. Calibration of relative humidity was undertaken using ASTM D5032, wherein a water/glycerol solution was prepared with the concentration required to achieve the desired relative humidity. This solution was added to Petri dishes and placed inside the chamber along with the LCES system. For a typical calibration, three Petri dishes each containing 50 ± 1 mL of solution were used. Following sealing, the chamber was purged with zero air at a 5 L/min flow rate for approximately 5 min. Relative humidity was then allowed to equilibrate and the final refractive index of the solution was used to determine the stabilized relative humidity value due to water evaporation or absorption into or from the airspace above the solution during the stabilization period.

A custom ABS, 3D printed frame was constructed to support the LCES. From a cost perspective, the above system provides significant capital expenditure savings compared to chemiluminescence detectors (Table 2, Figure 1b).

### 2.1. Software Control System

User control of the system was achieved through a graphical user interface written in C#. To enable the compiled code to run on the Raspbian operating system, Mono, a software framework built as an open-source and free alternative to the. NET Framework, was installed on the Raspberry Pi.

The C# program calls a series of python scripts that handle the interrogation of the attached sensors. Open-source python libraries were imported into the scripts at runtime to provide access to system functionality such as the ASD1115 bus and GPIO pins on the Raspberry Pi4. Data were written to comma-separated value (csv) files which were subsequently imported into Microsoft Excel for analysis. The data files were initially written to the micro-SD card inserted into the Raspberry Pi before being uploaded to a cloud storage account. Data were visualized in real time using an Adafruit IO cloud-based dashboard.

### 2.2. Control of NO Concentrations

NO at a mixing ratio of 60 ppm ±2% in nitrogen was obtained from CAC Gas and Instrumentation. A 0.5 L/min fixed flow rate regulator was attached to the NO cylinder. Gas was fed from this regulator to a calibrated Swagelok VAF-G2-01M-1-0 Variable Area Flowmeter which was set to deliver a 1.0 L/h flow rate. Gas exited the flowmeter and entered a 2-position, 3-way electrically controlled solenoid valve (refer Supplementary Materials) controlled from the microcomputer. The response time of the solenoid valve was approximately 30 ms.

At predetermined intervals, the solenoid valve was energized, and gas flow was diverted into the reaction chamber. Typically, at a 1 L/h flow rate of 60 ppmV ±2% NO gas, the solenoid valve required energizing for 9.444 s to deliver sufficient gas to increase the NO concentration in the chamber by 20 ppbV (refer Supplementary Materials for a typical electrode response to 20 ppbV stepwise nitric oxide addition and resultant calibration curve).

According to Alphasense, the NO electrochemical sensor has a response time (t90) of <25 s. To ensure that the sensor had stabilized following gas addition, a post stabilization time of 5 min was provided before the next gas addition.

The 30 ms response time of the solenoid valve, therefore, resulted in an error of ±0.064 ppbV under the conditions described above. This error is approximately 3.18% of the error associated with the stock gas concentration. In experiments with stepwise additions of NO gas, the total combined error associated with this addition method was ±0.464 ppbV per 20 ppbV step change. When a typical 100 ppbV, single-step gas addition was used, the total error was estimated to be ±2.064 ppbV, compared to ±2.320 ppbV for a multi-step addition.

## 3. Results

### 3.1. Electrode Output Stability

**Figure 2:**
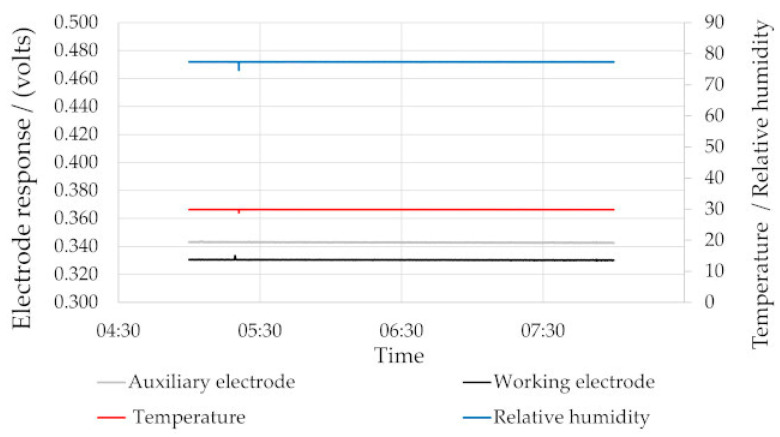
Stable electrochemical responses under isothermal/isohume conditions.

The output of LCESs, particularly NO sensors, are susceptible to variations in ambient environmental conditions [28]. When the environment can be precisely controlled, such as under isothermal/isohume conditions in a sealed chamber, stable outputs could be obtained from the LCESs (Figure 2).

In the latest generation four-electrode LCESs, such as those used during this study, the auxiliary electrode is isolated from the environment containing the target gas. Therefore, relative humidity fluctuations only affect the working electrode, leaving the responses from the auxiliary electrode unaltered.

**Figure 3:**
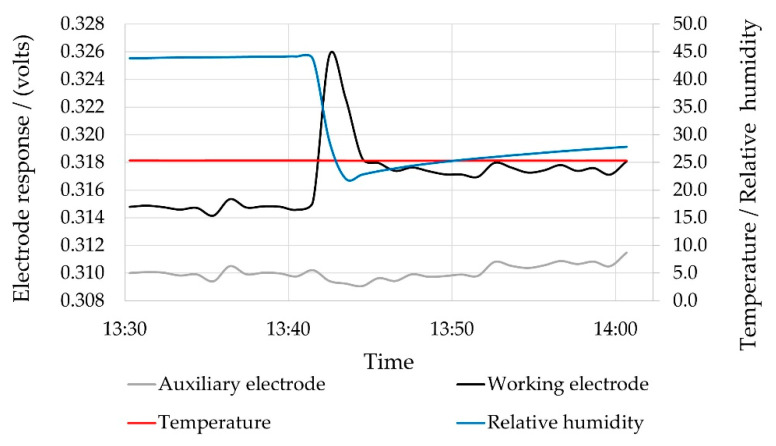
Working electrode responses to humidity transient.

Figure 3 demonstrates a scenario in which the chamber was purged with zero air and allowed to equilibrate before again purging with zero air at the 13:40 timestamp, to simulate a humidity transient. The result confirmed a transient response on the working electrode, which was exposed to the chamber environment, whereas the auxiliary electrode remained unaffected.

**Figure 4:**
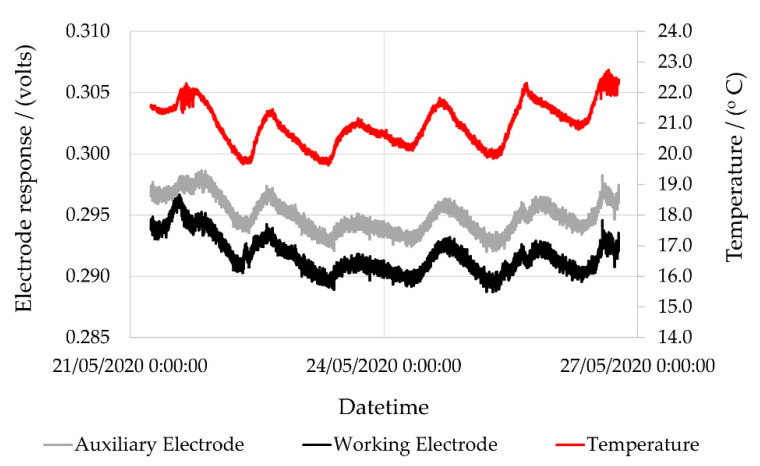
Temporal changes in NO electrochemical electrode responses due to changing temperature (dT = ±1.2 °C).

**Figure 5:**
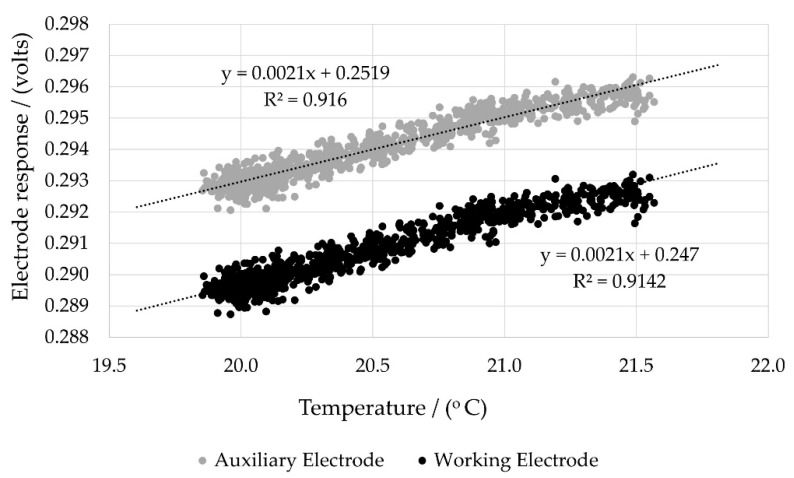
Temperature-dependence of NO electrochemical electrode responses (dT = ±1.2 °C).

Where temperature fluctuations occurred, both the working and auxiliary electrodes were affected, albeit to slightly different extents (Figure 4). From these data, the temperature dependence for the auxiliary and working electrode responses could be determined. Both the auxiliary and working electrodes displayed high correlation coefficients with temperature (Figure 5). This in turn allowed the interrelationship between the auxiliary and working electrode responses resulting from minor temperature fluctuations to be plotted.

**Figure 6:**
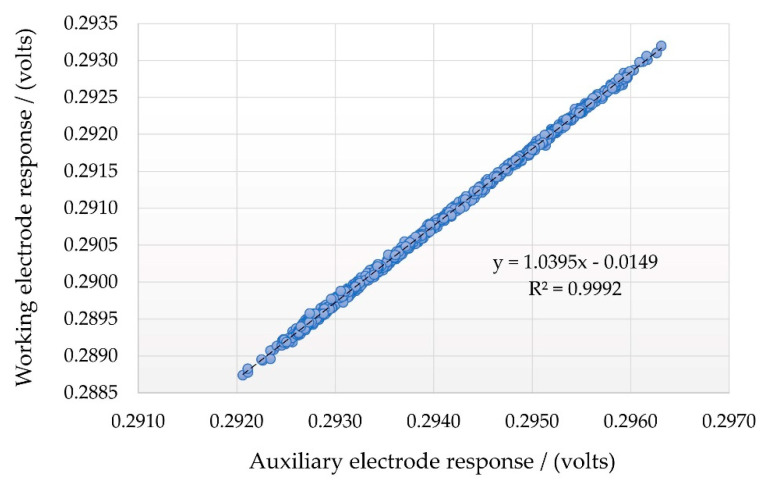
Interrelationship between NO electrodes to minor temperature fluctuations (dT = ±1.2 °C).

Figure 6 shows a highly linear interrelationship between the auxiliary and working electrodes over a minor temperature range variation. In this situation, chamber environmental conditions varied by approximately ±1.2 °C, and relative humidity was maintained at ±0.5%. After the temperature and auxiliary electrode responses were measured, the expected zero value for the working electrode response could be calculated.

**Figure 7:**
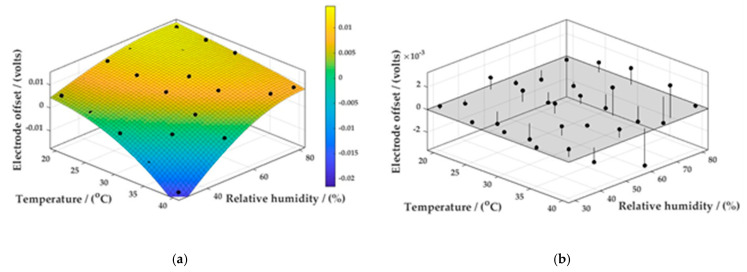
(**a**) MATLAB surface function of offset between working to auxiliary electrode across an environmental envelope. (**b**) Residuals plot of actual values to surface function.

Using this calculated zero air value for the working electrode response, the target gas concentration under the controlled conditions could be determined from the difference between the expected zero air baseline response at the current sensor temperature and the actual measured output, based on a calibration curve that had previously been determined under the same temperature and relativity humidity conditions. By maintaining isohume conditions and varying the chamber temperature, we determined that this linear relationship extended across a temperature range from 20 to 40 °C. The calculated gradient was greater than unity, indicating that the working electrode output response was increasing at a more rapid rate than that of the auxiliary electrode. When data from varying humidity’s were combined, it was also noted that whilst the linear relationship between the auxiliary and working electrode zero air responses was maintained, the gradient and intercept of each data series changed considerably, suggesting a polynomial rather than linear surface function (Table 3; Figure 7).

The surface function described by Figure 7 was obtained from the MATLAB Curve Fitting App using a third-degree polynomial fit (Poly33) in temperature and relative humidity. The function was of the following form:

(1) $$f\left(x,y\right)=p00+p10x+p01y+p20x^{2}+p11xy+p02y^{2}+p30x^{3}+p21x^{2}y+p12xy^{2}+p03y^{3}$$

where *x* is temperature, and *y* is relative humidity. The coefficients for the equation are reported in Table 4.

**Table 4:** Coefficients for Figure 8 (with 95% confidence bounds).

Coefficient	Value	95% Confidence Bounds
p00 =	−0.009984	(−0.09984, 0.07987)
p10 =	0.001054	(−0.006982, 0.009089)
p01 =	0.0001301	(−0.001954, 0.002215)
p20 =	−2.919 × 10^−5^	(−0.0002893, 0.0002309)
p11 =	−9.535 × 10^−6^	(−7.274 × 10^−5^, 5.367 × 10^−5^)
p02 =	7.243 × 10^−6^	(−2.478 × 10^−5^, 3.926 × 10^−5^)
p30 =	−5.231 × 10^−7^	(−3.363 × 10^−6^, 2.316 × 10^−6^)
p21 =	8.731 × 10^−7^	(2.827 × 10^−8^, 1.714 × 10^−6^)
p12 =	−2.413 × 10^−7^	(−5.828 × 10^−7^, 1.002 × 10^−7^)
p03 =	−3.849 × 10^−8^	(−2.218 × 10^−7^, 1.448 × 10^−7^)

The third-degree polynomial fit described by Equation (1) yielded an R^2^ value of 0.9697, a sum of squares due to error (SSE) value of 5.003 × 10^−5^, and a root mean square error (RMSE) of 0.001582. Higher degree polynomials did not notably improve the R^2^, SSE, or RMSE values. The residuals plot derived from the polynomial fit (Figure 9 showed higher deviations with the surface fit with increasing temperature and relative humidity conditions, supporting observations by Rogulski et al. [30].

### 3.2. Effect of Varying Environmental Conditions on NO Calibration Curves

Individual calibration curves using the method described previously were obtained across the environmental envelope of 20–40 °C and 30–80% relative humidity. Highly linear correlation coefficients were found for all the stepwise experiments (Table 5).

Several sets of data were obtained when calibration curves were prepared under isothermal/isohume conditions across an environmental envelope. Firstly, the electrode responses for both auxiliary and working electrodes could be determined for any NO concentration. Third-degree polynomial surface fits similar to those shown in Figure 7 could also be constructed from these data. More importantly, a third-degree polynomial surface fit of the form described by Equation (1), for the offset between the working and auxiliary electrode, could be constructed for these data for each measured environmental condition (Figure 7). This surface fit also displayed a high correlation, with an R^2^ value of 0.963, an SSE value of 6.213 × 10^−5^, and RMSE of 0.001762. The residual plot (not shown) demonstrated similar deviations from the surface fit with increasing temperature and relative humidity.

**Figure 8:**
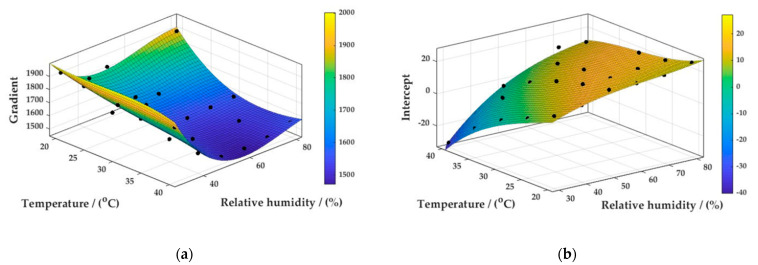
MATLAB surface function describing the gradients (**a**) and intercepts (**b**) resulting from the calibration curves measured across an environmental envelope.

**Figure 9:**
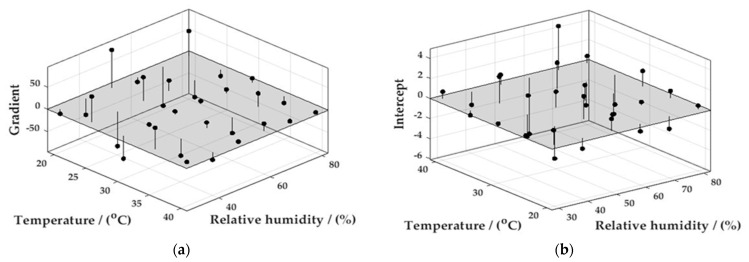
MATLAB residuals plots resulting from the calibration curves measured across an environmental envelope for, (**a**) Gradient and (**b**) Intercept.

Further surface fits could likewise be constructed for the gradients and intercepts of the calibration curves described above (Figure 8—Surface Fits, Figure 9—Residuals plot). 

R^2^ values of 0.9321 and 0.9741 were obtained for the gradient and intercept fits, respectively; SSE and RMSE values of 3.017 × 10^−4^ and 44.85, respectively, were obtained for the gradient fit, and of 122.48 and 2.4747, respectively, for the intercept fit.

### 3.3. Reproducibility and Transferability of Surface Functions

Having defined surface functions for the working to auxiliary electrode offset, and gradients and intercepts for calibration graphs, a series of experiments were conducted to determine the reproducibility with the original sensor and the transferability to new NOA4 sensors. Four environmental conditions with three NO concentrations were employed, and the results were calculated using both the initial calibration curve for the specific environmental condition and the gradient and intercept surface functions.

**Figure 10:**
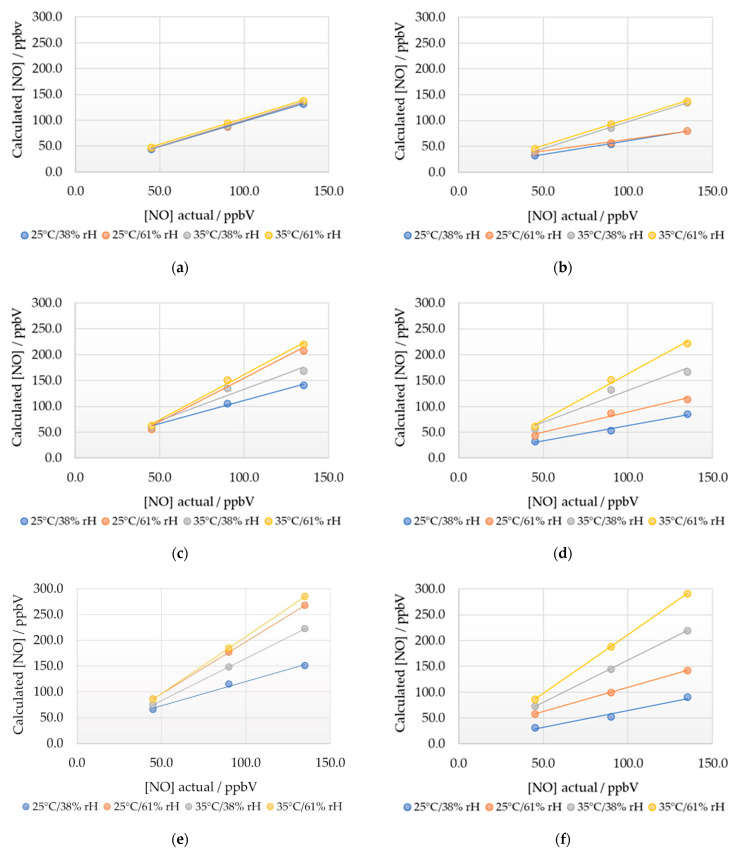
Calculated NO concentrations for three sensors under four different environmental conditions using the initially measured calibration curves, (**a**)—Original sensor, (**c**)—Sensor 2, (**e**)—(Sensor 3) and using the NO working electrode to NO auxiliary electrode offset, gradient, and intercept surface functions from the initially measured calibration curves (**b**)—Original sensor, (**d**)—Sensor 2, (**f**)—(Sensor 3).

The original sensor produced results within ±3% of the expected value using the calibration curve method suggesting that the initially constructed calibration curves had remained stable over a period of approximately three months (Figure 10a,b). Sensors 2 and 3, however, showed variation from expected values ranging from −40% to +115% of the known concentrations (Figure 10c–f). These variations typically increased under higher temperature and relative humidity. Higher levels of deviation from expected values resulted from the use of the surface functions. It is unclear if this was an artifact of the surface functions themselves, the small sample set of sensors, or a combination of both. Further work increasing the sample size of sensors and/or individual environmental conditions may assist in determining the cause of the deviation from expected NO values when using the surface functions. The results highlight that while the calibration for individual sensors is stable over monthly timescales, the calibration parameters are not transferable across sensors, and therefore the calibration procedure needs to be carried out for each sensor.

## 4. Conclusions

Electrochemical sensors, particularly NO sensors, are prone to variations in response due to variations in ambient environmental conditions. We demonstrated that under well controlled environmental conditions, i.e., isothermal/isohume conditions within a sealed environmental (reaction) chamber, stable responses can be achieved by NO electrochemical sensors. Furthermore, linear (although environmental-condition-dependent) calibration curves were obtained. Polynomial surface functions constructed from the individual linear calibration curves displayed good reproducibility under certain environmental conditions but resulted in increasing deviations under higher temperature and relative humidity conditions. Furthermore, we demonstrated that neither the linear nor the polynomial surface function calibration parameters could be transferred from the original sensors to alternate units.

Whilst the results reported in this paper are not immediately applicable to applications such as air quality monitoring without post-data corrections or use of machine learning algorithms, the linearity and reproducibility under isothermal/isohume conditions suggested that the sensors are suitable for applications where the experimental conditions are well controlled. Such applications would include NO kinetic studies for atmospheric applications, or the measurement of potential biocatalytic activity of NO -consuming enzymes in biocatalytic coatings, both of which currently employ high-capital-cost chemiluminescence detectors. We will focus on these areas in subsequent studies.

## Figures and Tables

**Table 1:** Composition of “zero air” used for purging environmental chamber.

Component	Composition (mole/mole)
Nitrogen	78.1%
Oxygen	20.9%
Argon	0.9%
Water	<25 ppm

**Table 2:** Breakdown of sensor system component costs.

Component	Cost (AUD)
Raspberry Pi 4 (4GB Ram)	105.00
Raspberry Pi 4 power supply	16.50
South Coast Science DFE	350.00
Alphasense two sensor AFE	204.00
Alphasense NO-A4 nitric oxide sensor	85.00
Alphasense Dummy sensor	0.00
Bosch BME280 sensor	7.00
Total	767.50

**Table 3:** Trendline equations and correlation coefficients for auxiliary and working electrode relationships across varying humidity’s.

Relative Humidity (%)	Trendline Equation	R^2^
30.0	y = 1.5361x − 0.1752	0.9999
40.0	y = 1.4274x − 0.1474	0.9977
50.0	y = 1.3356x − 0.1236	0.9987
60.0	y = 1.3282x − 0.1230	0.9808
70.0	y = 1.1845x − 0.0738	0.9815

**Table 5:** Correlation coefficients for calibration curves measured under isothermal/isohume conditions.

Relative Humidity/(%)	Temperature/(°C)				
	20.0	25.0	30.0	35.0	40.0
30.0	0.9980	0.9993	0.9995	0.9993	0.9991
40.0	0.9991	0.9997	0.9994	0.9996	0.9995
50.0	0.9998	0.9997	0.9998	0.9997	0.9997
60.0	0.9998	0.9997	0.9997	0.9998	0.9998
70.0	0.9999	0.9999	0.9998	0.9998	0.9999
80.0	0.9999	0.9996	0.9995	0.9997	0.9999

## Data Availability

The data presented in this study are available on request from the corresponding author. These data are not publicly available due to data collection format and authentication requirements of the current cloud storage location.

## Supplementary Materials

The following supporting information can be downloaded at: https://www.mdpi.com/article/10.3390/s22229013/s1, Figure S1: Two position, three-way electrically controlled solenoid valve; Figure S2: Typical electrode responses to 20 ppbV stepwise nitric oxide addition (chamber conditions 25 °C/50%rH); Figure S3: Typical calibration curve for 20 ppbV stepwise nitric oxide addition (chamber conditions 25 °C/50%rH); Figure S4: Nitric oxide auxiliary electrode (NOAE) surface function; Figure S5: Nitric oxide auxiliary electrode (NOAE) residuals plot; Table S1: Coefficients for Figure S4; Figure S6: Nitric oxide working electrode (NOWE) surface function; Figure S7: Nitric oxide working electrode (NOWE) residuals plot; Table S2: Coefficients for Figure S6; Table S3: Coefficients for Figure 8b; Table S4: Coefficients for Figure 9a; Table S5: Coefficients for Figure 9b.

## References

[1] Household Air Pollution and Health. https://www.who.int/news-room/fact-sheets/detail/household-air-pollution-and-health.

[2] Chau T.T., Wang K.Y. (2020). An association between air pollution and daily most frequently visits of eighteen outpatient diseases in an industrial city. Sci. Rep..

[3] Hagenbjörk-Gustafsson A., Lindahl R., Levin J.-O., Karlsson D. (1999). Validation of a diffusive sampler for NO_2_. J. Environ. Monit..

[4] Lewis A.C., Edwards P.M. (2016). Validate personal air-pollution sensors. Nature.

[5] Ayers G.P., Keywood M.D., Gillett R., Manins P.C., Malfroy H., Bardsley T. (1998). Validation of passive diffusion samplers for SO_2_ and NO_2_. Atmos. Environ..

[6] Hagenbjörk-Gustafsson A., Lindahl R., Levin J.O., Karlsson D. (2002). Validation of the Willems badge diffusive sampler for nitrogen dioxide determinations in occupational environments. Analyst.

[7] Ionov D., Poberovskii A. (2015). Quantification of NOx emission from St Petersburg (Russia) using mobile DOAS measurements around the entire city. Int. J. Remote Sens..

[8] Wojtas J., Mikolajczyk J., Bielecki Z. (2013). Aspects of the application of cavity enhanced spectroscopy to nitrogen oxides detection. Sensors.

[9] Brown S.S., An H., Lee M., Park J.-H., Lee S.-D., Fibiger D.L., McDuffie E.E., Dubé W.P., Wagner N.L., Min K.-E. (2017). Cavity enhanced spectroscopy for measurement of nitrogen oxides in the Anthropocene: Results from the Seoul tower during MAPS 2015. Faraday Discuss..

[10] Kipping P.J., Jeffery P.G. (1963). Detection of Nitric Oxide by Gas-chromatography. Nature.

[11] Funazo K., Tanaka M., Shono T. (1978). New Gas Chromatographic Detection of Nitric Oxide. Anal. Lett..

[12] Smith R.N., Swinehart J., Lesnini D.G. (1958). Chromatographic Analysis of Gas Mixtures Containing Nitrogen, Nitrous Oxide, Nitric Oxide, Carbon Monoxide, and Carbon Dioxide. Anal. Chem..

[13] Pan S., Tian Y., Li M., Zhao J., Zhu L., Zhang W., Gu H., Wang H., Shi J., Fang X. (2015). Quantitative detection of nitric oxide in exhaled human breath by extractive electrospray ionization mass spectrometry. Sci. Rep..

[14] Campau R.M., Neerman J.C. (1967). Continuous Mass Spectrometric Determination of Nitric Oxide in Automotive Exhaust. SAE Trans..

[15] Venables D.S., Guardia M.A.S. (2016). Spectroscopic Measurement of Pollutant Gases. Comprehensive Analytical Chemistry.

[16] Department of Planning Industry and Environment NSW Compliance Report 2020. National Environment Protection (Ambient Air Quality) Measure 2021. https://www.environment.nsw.gov.au/research-and-publications/publications-search/new-south-wales-annual-compliance-report-2020.

[17] (2011). Methods for Sampling and Analysis of Ambient Air Determination of Oxides of Nitrogen—Direct-Reading Instrumental Method.

[18] (2012). Ambient Air. Standard Method for the Measurement of the Concentration of Nitrogen Dioxide and Nitrogen Monoxide by Chemiluminescence.

[19] Australian Government National Environment Protection (Ambient Air Quality) Measure. https://www.legislation.gov.au/Details/F2021C00475.

[20] Brown V.R. (2006). Chapter 6:Training Articles: Hand-held portable gas detectors. The Grey House Safety and Security Directory.

[21] Mead M.I., Popoola O.A.M., Stewart G.B., Landshoff P., Calleja M., Hayes M., Baldovi J.J., McLeod M.W., Hodgson T.F., Dicks J. (2013). The use of electrochemical sensors for monitoring urban air quality in low-cost, high-density networks. Atmos. Environ..

[22] Gill A., Zajda J., Meyerhoff M.E. (2019). Comparison of electrochemical nitric oxide detection methods with chemiluminescence for measuring nitrite concentration in food samples. Anal. Chim. Acta.

[23] Lewis A.C., Lee J.D., Edwards P.M., Shaw M.D., Evans M.J., Moller S.J., Smith K.R., Buckley J.W., Ellis M., Gillot S.R. (2016). Evaluating the performance of low cost chemical sensors for air pollution research. Faraday Discuss..

[24] Cross E.S., Williams L.R., Lewis D.K., Magoon G.R., Onasch T.B., Kaminsky M.L., Worsnop D.R., Jayne J.T. (2017). Use of electrochemical sensors for measurement of air pollution: Correcting interference response and validating measurements. Atmos. Meas. Tech..

[25] Kamionka M., Breuil P., Pijolat C. (2006). Calibration of a multivariate gas sensing device for atmospheric pollution measurement. Sens. Actuators B Chem..

[26] Malings C., Tanzer R., Hauryliuk A., Kumar S.P.N., Zimmerman N., Kara L.B., Presto A.A., Subramanian R. (2019). Development of a general calibration model and long-term performance evaluation of low-cost sensors for air pollutant gas monitoring. Atmos. Meas. Tech..

[27] Zimmerman N., Presto A.A., Kumar S.P.N., Gu J., Hauryliuk A., Robinson E.S., Robinson A.L., Subramanian R. (2018). A machine learning calibration model using random forests to improve sensor performance for lower-cost air quality monitoring. Atmos. Meas. Tech..

[28] Popoola O.A.M., Stewart G.B., Mead M.I., Jones R.L. (2016). Development of a baseline-temperature correction methodology for electrochemical sensors and its implications for long-term stability. Atmos. Environ..

[29] Wei P., Ning Z., Ye S., Sun L., Yang F., Wong K.C., Westerdahl D., Louie P. (2018). Impact Analysis of Temperature and Humidity Conditions on Electrochemical Sensor Response in Ambient Air Quality Monitoring. Sensors.

[30] Rogulski M., Badyda A., Gayer A., Reis J. (2022). Improving the Quality of Measurements Made by Alphasense NO_2_ Non-Reference Sensors Using the Mathematical Methods. Sensors.

