# Supplementary material for: Low-Cost Nitric Oxide Sensors: Assessment of Temperature and Humidity Effects
Source: Sensors (Basel). 2022 Nov 21;22(22):9013. doi: 10.3390/s22229013 (PMC9699606; doi:10.3390/s22229013)
Supplement: Supplementary file 1 [file sensors-22-09013-s001.zip › sensors-1870495-supplementary.docx]

Supplementary Material

**Low-cost nitric oxide sensors: Assessment of temperature and humidity effects**

**Steven Owen ^1,^*, Lachlan Yee ^2^ and Damien Maher ^2^**

^1^ Illawarra Coatings, 19 Technology Drive, Appin, NSW 2560, Australia

^2^ Faculty of Science and Engineering, Southern Cross University, Military Road,
Lismore, NSW 2480, Australia

***** Correspondence: steven@illawarracoatings.com


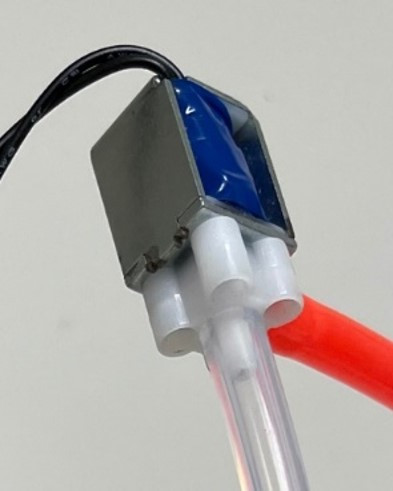


Figure S1: Two position, three way electrically controlled solenoid valve


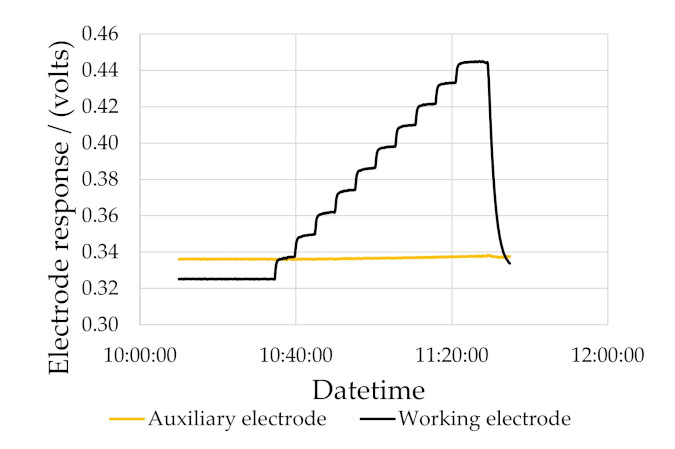


Figure S2: Typical electrode responses to 20 ppbV stepwise nitric oxide addition (chamber conditions 25 °C/50% relative humidity).

| 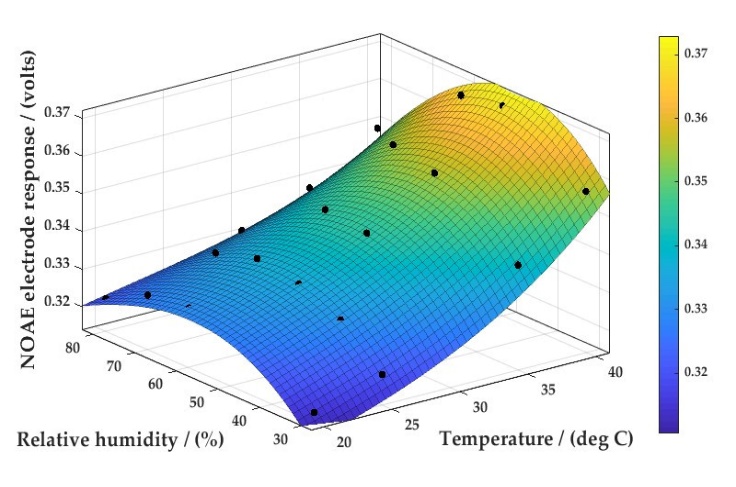 Figure S4: Nitric oxide auxiliary electrode surface function | 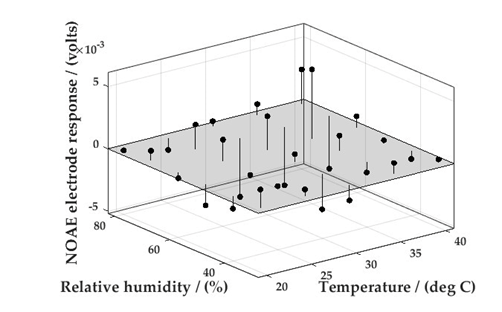 Figure S5: Nitric oxide auxiliary electrode residuals plot for Figure S4. |
| --- | --- |
|  | \| Table S1. Coefficients for Figure S4. \| \| \| \| --- \| --- \| --- \| \| p00 =  p10 =  p01 =  p20 =  p11 =  p02 =  p30 =  p21 =  p12 =  p03 = \| 0.2386  0.0010025  0.002453  -6.782 × 10^−5^  6.537 × 10**^−^**^5^  -3.581 × 10**^−^**^5^  1.601 × 10**^−6^**  -5.057 × 10**^−7^**  -4.76 × 10**^−7^** \| (-0.08908, 0.388)  (-0.01234, 0.01439)  (-0.001014, 0.00592)  (-0.0005005, 0.0003649)  (-3.976 × 10**^−^**^5^, 0.0001705)  (-8.907 × 10**^−^**^5^, 1.746 × 10**^−^**^5^)  (-3.122 × 10**^−6^**, 6.324 × 10**^−6^**)  (-1.91 × 10**^−6^**, 8.949 × 10**^−7^**)  (-1.044 × 10**^−6^**, 9.203 × 10**^−6^**)  (-1.423 × 10**^−7^**, 4.674 × 10**^−7^**) \| |


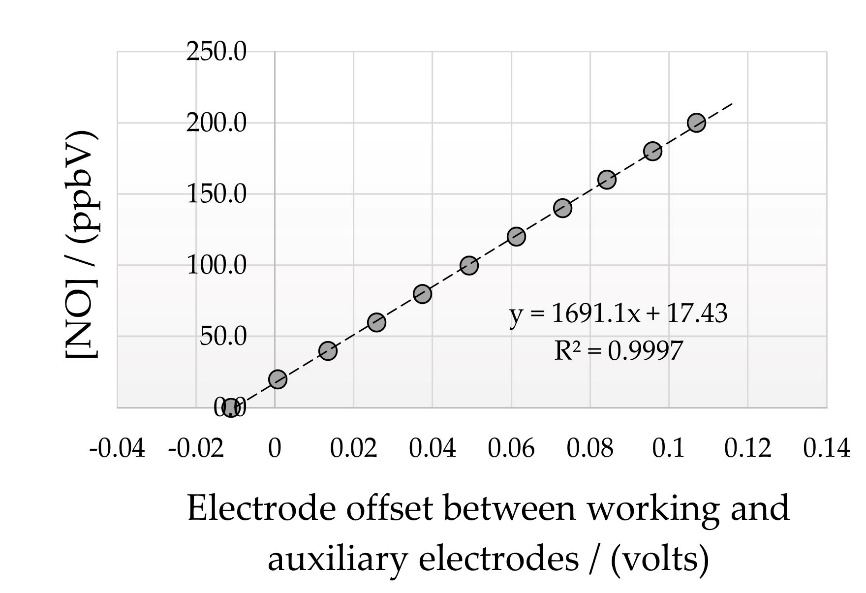


Figure S3: Typical calibration curve for 20 ppbV stepwise nitric oxide addition (chamber conditions 25 °C/50% relative humidity).

| 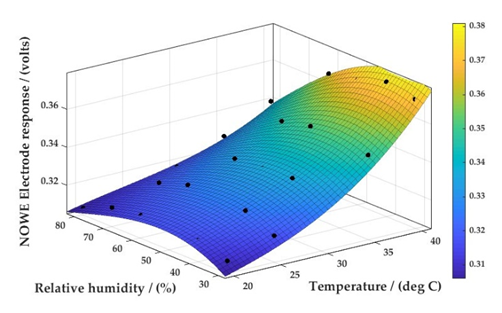 Figure S6: Nitric oxide working electrode surface function | 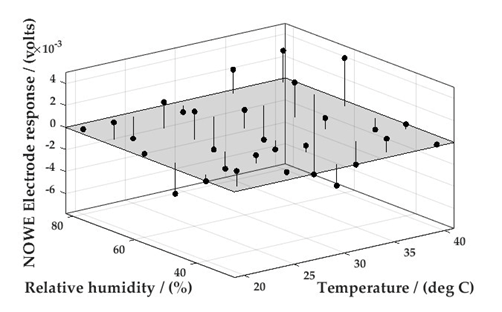  Figure S7: Nitric oxide working electrode residuals plot for Figure S6 |
| --- | --- |
|  | \| Table S2. Coefficients for Figure S6. \| \| \| \| --- \| --- \| --- \| \| p00 =  p10 =  p01 =  p20 =  p11 =  p02 =  p30 =  p21 =  p12 =  p03 = \| 0.2348  23B0.001371  24B0.002362  25B-8.706 × 10^−5^  7.532 × 10^−^**^5^**  -4.378 × 10^−^**^5^**  2.626 × 10^−^**^6^**  -1.312 × 10^−^**^7^**  -2.743 × 10^−^**^7^**  2.106 × 10^−^**^7^** \| (0.07281, 0.3967)  (-0.01311, 0.01585)  (-0.001395, 0.006119)  (-0.0005559, 0.0003818)  (-3.86 × 10^−5^, 0.0001892)  (-0.0001015, 1.39 × 10^−^**^5^**)  (-2.492 × 10^−^**^5^**, 7.74 × 10^−6^)  (-2.832 × 10^−6^, 2.075 × 10^−7^)  (-8.898 × 10^−7^, 3.412 × 10^−7^)  (-1.197 × 10**^−^**^7^, 5.41 × 10^−7^) \| |

| \| Table S3. Coefficients for Figure 8a. \| \| \| \| --- \| --- \| --- \| \| 26Bp00 =  27Bp10 =  28Bp01 =  29Bp20 =  30Bp11 =  31Bp02 =  32Bp30 =  33Bp21 =  34Bp12 =  p03 = \| -0.003784  0.0003459  -9.039 × 10**^−^**^5^  -1.924 × 10**^−^**^5^  9.943 × 10**^−^**^6^  -7.973 × 10**^−^**^6^  1.024 × 10**^−^**^6^  -8.046 × 10**^−^**^7^  2.017 × 10**^−^**^7^  4.804 × 10**^−^**^8^ \| 35B(-0.1039, 0.09635)  36B(-0.008609, 0.009301)  37B(-0.002413, 0.002232)  38B(-0.0003091, 0.0002706)  39B(-6.049 × 10^−5^, 8.038 × 10^−5^)  40B(-4.366 × 10^−5^, 2.771 × 10^−5^)  41B(-2.14 × 10^−6^, 4.189 × 10^−6^)  42B(-1.744 × 10^−6^, 1.349 × 10^−7^)  43B(-1.788 × 10^−7^, 5.823 × 10^−7^)  44B(-1.562× 10^−7^, 2.523× 10^−7^) \| | | |  | \| Table S4. Coefficients for Figure 9a. \| \| \| \| --- \| --- \| --- \| \| 45Bp00 =  46Bp10 =  47Bp01 =  48Bp20 =  49Bp11 =  50Bp02 =  51Bp30 =  52Bp21 =  53Bp12 =  p03 = \| 54B2696  55B-3.785  56B-37.56  57B1.838  -2.749  1.142  -0.03087  0.02883  0.007405  -0.006482 \| (303.3, 5636)  (-242.2, 234.6)  (-99.41, 24.29)  (-5.88, 9.556)  (-4.642, -0.8735)  (0.1922, 2.092))  (-0.1151, 0.05338)  (0.003811, 0.05384)  (-0.002728, 0.01754)  (-0.01192, -0.001044) \| | | |
| --- | --- | --- | --- | --- | --- | --- | --- | --- | --- | --- | --- | --- | --- | --- | --- | --- | --- | --- |
|  |  |  |  |  |  |  |
|  |  |  |  |  |  |  |
|  | | | | \| Table S5. Coefficients for Figure 9b. \| \| \| \| --- \| --- \| --- \| \| 58Bp00 =  59Bp10 =  60Bp01 =  61Bp20 =  62Bp11 =  63Bp02 =  64Bp30 =  65Bp21 =  66Bp12 =  p03 = \| 67B34.4  68B1.271  69B-4.532  70B-0.0144  71B-0.001114  72B0.1303  73B0.0001344  74B-0.0005955  75B0.001585  -0.00273 \| 76B(-106.2, 175)  77B(-1.991, 4.532)  78B(-17.11, 8.042)  79B(-0.0645, 0.0357)  80B(-0.1, 0.09778)  81B(-0.2767, 0.5373)  82B(-0.0001524, 0.0004211)  83B(-0.00113, -6.114 × 10^−5^)  84B(0.0002659, 0.002904)  (-0.007173, 0.001713) \| | | |
